# Recombinant Herpesvirus Glycoprotein G Improves the Protective Immune Response to *Helicobacter pylori* Vaccination in a Mouse Model of Disease

**DOI:** 10.1371/journal.pone.0096563

**Published:** 2014-05-02

**Authors:** Louise Baker, Andre M. L. Chitas, Carol A. Hartley, Mauricio J. C. Coppo, Paola K. Vaz, Andrew Stent, James R. Gilkerson, Joanne M. Devlin, Alison L. Every

**Affiliations:** 1 Centre for Animal Biotechnology, Faculty of Veterinary Science, The University of Melbourne, Parkville, VIC, Australia; 2 Asia-Pacific Centre for Animal Health, Faculty of Veterinary Science, The University of Melbourne, Parkville, VIC, Australia; 3 Centre for Equine Infectious Disease, Faculty of Veterinary Science, The University of Melbourne, Parkville, VIC, Australia; University of Liverpool, United Kingdom

## Abstract

Alphaherpesviruses, which have co-evolved with their hosts for more than 200 million years, evade and subvert host immune responses, in part, by expression of immuno-modulatory molecules. Alphaherpesviruses express a single, broadly conserved chemokine decoy receptor, glycoprotein G (gG), which can bind multiple chemokine classes from multiple species, including human and mouse. Previously, we demonstrated that infection of chickens with an infectious laryngotracheitis virus (ILTV) mutant deficient in gG resulted in altered host immune responses compared to infection with wild-type virus. The ability of gG to disrupt the chemokine network has the potential to be used therapeutically. Here we investigated whether gG from ILTV or equine herpesvirus 1 (EHV-1) could modulate the protective immune response induced by the *Helicobacter pylori* vaccine antigen, catalase (KatA). Subcutaneous immunisation of mice with KatA together with EHV-1 gG, but not ILTV gG, induced significantly higher anti-KatA IgG than KatA alone. Importantly, subcutaneous or intranasal immunisation with KatA and EHV-1 gG both resulted in significantly lower colonization levels of *H. pylori* colonization following challenge, compared to mice vaccinated with KatA alone. Indeed, the lowest colonization levels were observed in mice vaccinated with KatA and EHV-1 gG, subcutaneously. In contrast, formulations containing ILTV gG did not affect *H. pylori* colonisation levels. The difference in efficacy between EHV-1 gG and ILTV gG may reflect the different spectrum of chemokines bound by the two proteins. Together, these data indicate that the immuno-modulatory properties of viral gGs could be harnessed for improving immune responses to vaccine antigens. Future studies should focus on the mechanism of action and whether gG may have other therapeutic applications.

## Introduction

Alphaherpesviruses are a family of large double stranded DNA viruses that cause disease in humans and animals. Herpesviruses have been co-evolving with their hosts for more than 200 million years and utilize many strategies to subvert host immune responses, including decoy receptors that bind chemokines and modulate host responses to infection [1]–[3]. Alphaherpesviruses have only one broadly conserved chemokine decoy receptor, glycoprotein (gG). Glycoprotein G from most alphaherpesviruses function as broad-spectrum viral chemokine binding proteins (vCKBPs) *in vitro* [4]–[7]. Chemokines are small molecular mass cytokines that recruit and activate cells of the immune system. The chemokine network is a major target for immune-modulating drugs [8]–[12]. Glycoprotein G from diverse alphaherpesviruses bind a broad range of chemokine classes from several different species, including human and mouse species [4], [5], [7]. The chemokine-binding specificity of the gGs from at least 7 different herpesviruses have been studied and bind to a broad range of chemokines, including human and mouse CC and CXC chemokines. All gG homologues tested (except gG from equine herpesvirus 4) bind human CXCL11, 12, 13 and 14, and show variable binding to many other human CCL, CXCL, and CL chemokines [4]–[7], [13], [14].

Our previous studies of gG have made extensive use of avian infectious laryngotracheitis virus (ILTV) infection experiments in the natural host (chickens). This virus causes acute respiratory disease in poultry worldwide. Our *in vivo* studies have shown that gG modulates the inflammatory response and consequent adaptive immune response to alphaherpesvirus infection. In infection studies using wild-type and gG-deficient constructs of ILTV, the presence of gG results in increased recruitment of B cells and reduced recruitment of T cells and heterophils (avian neutrophil equivalent) to the site of infection [7]. Consistent with this finding, infection with gG-deficient ILTV results in significantly reduced serum and tracheal antibody titres to ILTV, yet can induce protective immunity from challenge with virulent ILTV [7], [15]–[17]. Animal models of human diseases have demonstrated therapeutic applications of other vCKBPs (from poxviruses) to alter host immune responses and reduce inflammation [18], [19], however the therapeutic use of gG has not been explored. As gG has no amino acid similarity with other vCKBPs, or with host chemokine receptors [5], this glycoprotein represents a novel protein structure that has the potential to be used therapeutically to influence the chemokine network. This study aims to explore whether recombinant gG from either a mammalian herpesvirus (equine herpesvirus 1, EHV-1) or avian herpesvirus (ILTV) can modulate the immune response to heterologous antigen using vaccination against the Gram negative human stomach pathogen, *Helicobacter pylori*, as a model.

*Helicobacter pylori* is one example of a bacterial infection for which many have attempted to develop a vaccine, with minimal success thus far [20]. Several human clinical trials have tested urease as the antigen, delivered in combination with the adjuvant, heat-labile toxin from *Escherichia coli* [21]–[23]. In one study, a significant reduction in bacterial load was noted, which was accompanied by induction of anti-*H. pylori* serum IgA and anti-*H. pylori* peripheral blood mononuclear antibody-secreting cells (ASCs) [21], although more than half the participants developed diarrhoea. To circumvent toxicity issues associated with LT, urease expressed by a *Salmonella enterica* serovar Typhi [24] or serovar Typhimurium [25] have been tested in clinical trials and were shown to induce anti-*H. pylori* immunity, with the latter study also noting reduced bacterial loads. Strong immune responses were also noted in a trial where participants were vaccinated via the intramuscular route with a combination of three *H. pylori* antigens, namely, VacA, CagA and NAP, adjuvanted with alum [26]. Although the approaches used thus far show promise, clinical trials demonstrating robust protective outcomes have not yet been reported. The two major obstacles that are yet to be overcome are: (i) induction of sterilising immunity and, (ii) identification of a non-toxic but effective adjuvant. In this study we tested whether glycoprotein G derived from EHV-1 or ILTV could be used to modulate immune responses to recombinant *H. pylori* catalase and whether this would lead to protective immunity against *H. pylori* challenge.

## Materials and Methods

### Baculovirus Expression of Recombinant EHV-1 and ILTV gG

Recombinant EHV-1 gG with a C-terminal histidine tag was expressed using the Bac-to-Bac Baculovirus expression system (GIBCO BRL, Carlsbad, CA). The required EHV-1 gG sequence was amplified from EHV-1.438/77 [27] DNA using primers (Geneworks, Hindmarsh, Australia) targeting the gG gene of EHV-1 (5′-TATTTAGGATCCATGTTGACTGTCTTAGCA-3′ and 5′-ATAATAGTCGAC *ATGATGATGATGATGGTG*CTGGATGCCGTTCGACGC-3′). The primer sequences included sites for restriction nuclease digestion (underlined) as well as nucleotides encoding a polyhistidine tag (italicized). The PCR product was ligated into the p*fastbac* vector (GIBCO BRL) after restriction endonuclease digestion of the vector and PCR product. The sequence was verified using Big Dye Terminator 3.1 chemistry (Applied Biosystems, Carlsbad, CA). After verification of the expected sequence, plasmids were then transformed into DH10Bac1 cells (Life Technologies, Carlsbad, CA) generating recombinant bacmid DNA encoding EHV-1 gG with a histidine tag. Sf9 insect cells were transfected with recombinant bacmid DNA using Cellfectin (Invitrogen, Carlsbad, CA), which were subsequently used to generate recombinant baculovirus particles expressing the EHV-1 gG.

Expression of EHV-1 gG from recombinant baculovirus infected Sf9 cells was confirmed after separation of proteins by SDS-PAGE, transfer to polyvinylidene difluoride (PVDF) membrane (Immobilon-P Transfer membrane, Millipore, Billerica, MA) and Western blot with hyperimmune rat serum to the variable region of EHV-1 gG [28]. Bound antibody was detected with horseradish peroxidase (HRP)-conjugated goat anti-rat IgG (GE Healthcare, Little Chalfont, Buckinghamshire, United Kingdom), ECL™ western blotting detection system (GE Healthcare) and autoradiography. Large scale cultures of infected Sf9 cells were prepared and recombinant gG was purified from Sf9 supernatant 72 hours after infection, with Ni-NTA agarose beads (Qiagen, Germantown, MD) after dialysis of the supernatant in lysis buffer (50 mM NaH_2_PO_4_, 300 mM NaCl, 10 mM imidazole, pH 8.0). Purity was determined by separation of proteins by SDS-PAGE and subsequent staining with Coomassie blue. Western blot was used to confirm the identity of the purified protein, as described above. The protein concentration was determined by Bradford assay (BioRad, Hercules, CA).

Recombinant ILTV gG was synthesised and purified using the same baculovirus expression system as EHV-1 gG, as previously described [7]. Previous studies have demonstrated the ability of the purified baculovirus-expressed ILTV gG to bind to chemokines [7].

### Chemokine-binding Enzyme-Linked Immunosorbent Assay (ELISA) Using EHV-1 gG

To assess the chemokine binding activity of recombinant EHV-1 gG, flat bottom microtitre plate wells (Nunc Polysorb, Penfield, NY, USA) were coated with dilutions of recombinant chemokine, in coating buffer (16 mM Na_2_CO_3_, 34 mM NaHCO_3,_ pH 9.6), beginning at a concentration of 6 µg/mL. Human IL-8 was used as a positive control for binding. Plates were covered and incubated overnight at 4°C prior to washing with PBS containing 0.05% v/v Tween 20 (PBS-T), and blocking for 2 hrs at 37°C with 10 mg/mL bovine serum albumin (BSA) in PBS (PBS-BSA) containing 5% v/v normal sheep serum. Plates were washed with PBS-T and incubated for 2 hrs at room temperature (RT) with EHV-1 gG (3 µg/mL) in BSA diluent (5 mg/ml BSA, PBS-T, 2.5% v/v normal sheep serum). Following washing with PBS-T, plates were incubated with a 1/100 dilution of hyperimmune rat serum to the variable region of EHV-1 gG [28] for 2 hrs at RT. Plates were washed and incubated for 1 hr with HRP-conjugated goat anti-rat Ig secondary antibody at RT. The plates were washed and developed using tetramethylbenzidine substrate (0.1 mg/mL, Sigma-Aldrich, St Louis, MO, USA) in developing buffer (0.1 M Na_2_HPO_4_, 0.05 M citric acid, pH 5.0, 0.006% v/v H_2_O_2_), and the reaction stopped by adding 1 M HCl. Absorbance readings with backgrounds subtracted (derived from wells containing no gG) were determined at a wavelength of 450 nm (Labsystems MultiskanMS).

### Mice

BALB/c mice were purchased from The Walter & Eliza Hall Institute and maintained in the Animal House Facility at Veterinary Science, University of Melbourne. All procedures were subject to approval from the University of Melbourne Animal Ethics Committee in accordance with the Australian Code of Practice for the care and use of animals for scientific purposes.

### *H. Pylori* Culture

*H. pylori* strain SS1 [29] was grown on horse blood agar plates and sub-cultured into brain heart infusion broth as described [30].

### *Helicobacter Pylori* Vaccination/Challenge

Recombinant, his-tagged *Helicobacter pylori* catalase (KatA) was synthesised and purified over Ni-NTA columns as described [31] and used in vaccination/challenge studies. Mice (n = 8) were vaccinated twice, three weeks apart as outlined in Table 1. Two additional groups were left unvaccinated. Four weeks after the second vaccination mice were challenged with *H. pylori* (10^7^ CFU) via oro-gastric gavage. Mice were euthanised four weeks after infection for assessment of bacterial burden by colony-forming unit assay as described [30].

**Table 1:** Vaccine formulations delivered to BALB/c mice.

Route	Vaccine antigen	Adjuvant	n
Subcutaneous	KatA	-	8
	Formalin-fixed *H. pylori*	-	8
	KatA	EHV-1 or ILTV gG^a^	8
Intranasal	KatA	-	8
	KatA	CT^b^	8
	KatA	EHV-1 or ILTV gG	8

a gG, glycoprotein G;

b CT, cholera toxin.

### Determination of Serum IgG Titre

Sera were collected by cardiac puncture and anti-*Helicobacter pylori* catalase antibody levels determined by standard direct ELISA as described [30]. Briefly, Maxisorp immunoplates (Nunc, Roskilde, Denmark) were coated overnight with 0.25 µg recombinant KatA in bicarbonate buffer pH 9.6. After blocking with PBS–BSA serial dilutions of sera were added to duplicate wells. After washing, HRP-conjugated goat anti-mouse IgG Fc (Pierce, Rockford, IL; diluted 1∶5000 in PBS–BSA) was added. Colour was developed by addition of 100 µL 3,3′5,5-tetramethylbenzidine (Invitrogen, Carlsbad, CA), and the reaction was stopped by adding 2 M H_2_SO_4_. Absorbance was read at 450 nm and mid-point titres calculated.

### Assessment of Gastritis

Gastritis was assessed histologically as described [32]. Briefly, half stomachs were fixed in 10% neutral-buffered formalin, processed, and embedded in paraffin wax. Sections (4 µm) were stained with haematoxylin and eosin and histopathology was scored by a blinded assessor according to the following scheme Cellular infiltrate 0–6: 0, none; 1 mild, multifocal; 2 mild, widespread or moderate multifocal; 3, mild widespread and moderate multifocal or severe multifocal; 4, moderate widespread; 5, moderate widespread and severe multifocal; and 6, severe widespread. Mucus metaplasia and functional atrophy 0–3: 0, absent; 1, mild; 2, moderate; and 3, severe.

### Statistical Analysis

One-way analysis of variance (ANOVA) with Dunnett’s Multiple Comparison Test was used to assess differences in gastritis development. Kruskal-Wallis one-way ANOVA with Dunn’s multiple comparison test was used to analyse ELISA and CFU data. All analysis was performed using GraphPad Prism software (Version 5, San Diego, CA, USA). A p-value of less than 0.05 was considered to indicate statistical significance.

## Results

### Synthesis and Purification of gG

**Figure 1:**
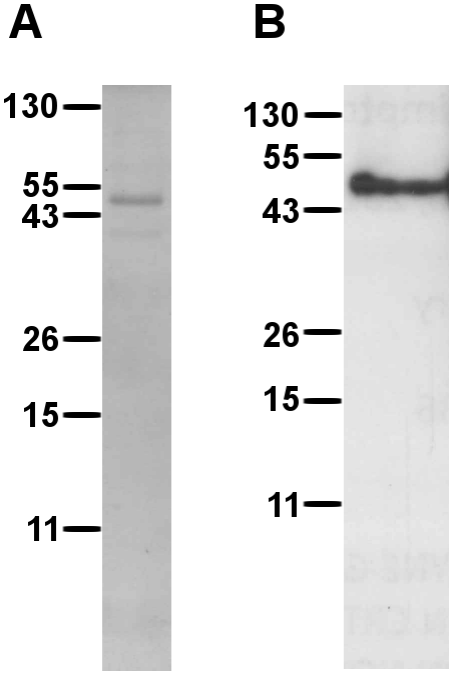
Recombinant EHV-1 gG purified from a baculovirus expression system. Purified EHV-1 gG from Sf9 cells infected with recombinant baculovirus, separated on 10% SDS-PAGE and (A) stained with Coomassie Brilliant Blue-250, or (B) transferred to PVDF membrane and probed with anti-EHV-1 gG rat polyclonal serum and HRP-conjugated goat anti-rat IgG.

Recombinant, purified EHV-1gG was separated by SDS-PAGE and stained with Coomassie Brilliant Blue (Figure 1A). Western blots were probed with EHV-1 gG specific polyclonal antiserum, and detected an approximately 48 kDa protein consistent with the band size observed in Coomassie Brilliant Blue stains (Figure 1B). This is consistent with the expected size of this glycoprotein, consisting of a 39.3 kDa amino acid backbone and 6 potential N-linked glycosylation sites. Results from Coomassie Brilliant Blue staining and Western blotting of recombinant, purified ILTV gG following SDS-PAGE were consistent with those described in previous studies [7] (not shown).

### EHV-1 gG Binds CCL5, CCL11 and the Th2 Cytokine, IL-4

**Figure 2:**
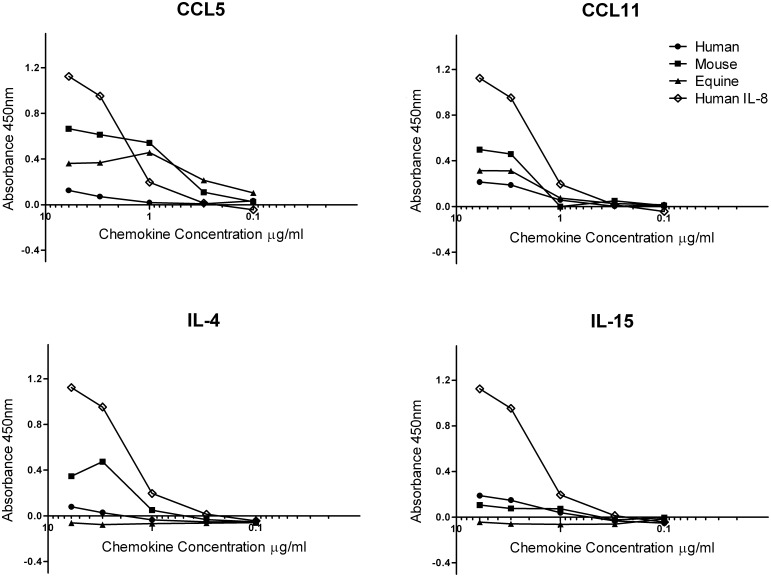
Chemokine-binding profiles of EHV-1 gG. Baculovirus expressed and 6xHis-tag purified EHV-1 gG was incubated with either human, mouse or equine chemokine- or cytokine-coated ELISA wells, before detection with EHV-1 gG-specific antibody. Human IL-8 is used as a positive control for gG binding. Absorbances were measured at 450 nm and backgrounds were subtracted (absorbance in wells containing no gG).

Chemokine-binding ELISAs utilised in this study yielded results consistent with previous studies [5], [13], [33] and showed that recombinant EHV-1 gG binds to mXCL1 and eIL-8 with the highest binding levels to hIL-8 and no binding to hCX_3_CL1 and eCCL2 (not shown). In addition, recombinant EHV-1 gG bound CCL5 and CCL11 (human, murine, equine) with some variation in binding observed between chemokines from the different species of origin (Figure 2). EHV-1 gG bound only to mouse CCL11 and poorly, if at all, to equine or human CCL11 (Figure 2). While EHV-1 gG bound the Th2 cytokine, IL-4 (murine), it failed to bind human and equine IL-4 or any species of the T-cell proliferative and memory maintenance cytokine, IL-15.

### EHV-1 gG, but not ILTV gG, Boosts Immunity to *H. Pylori* Catalase

**Figure 3:**
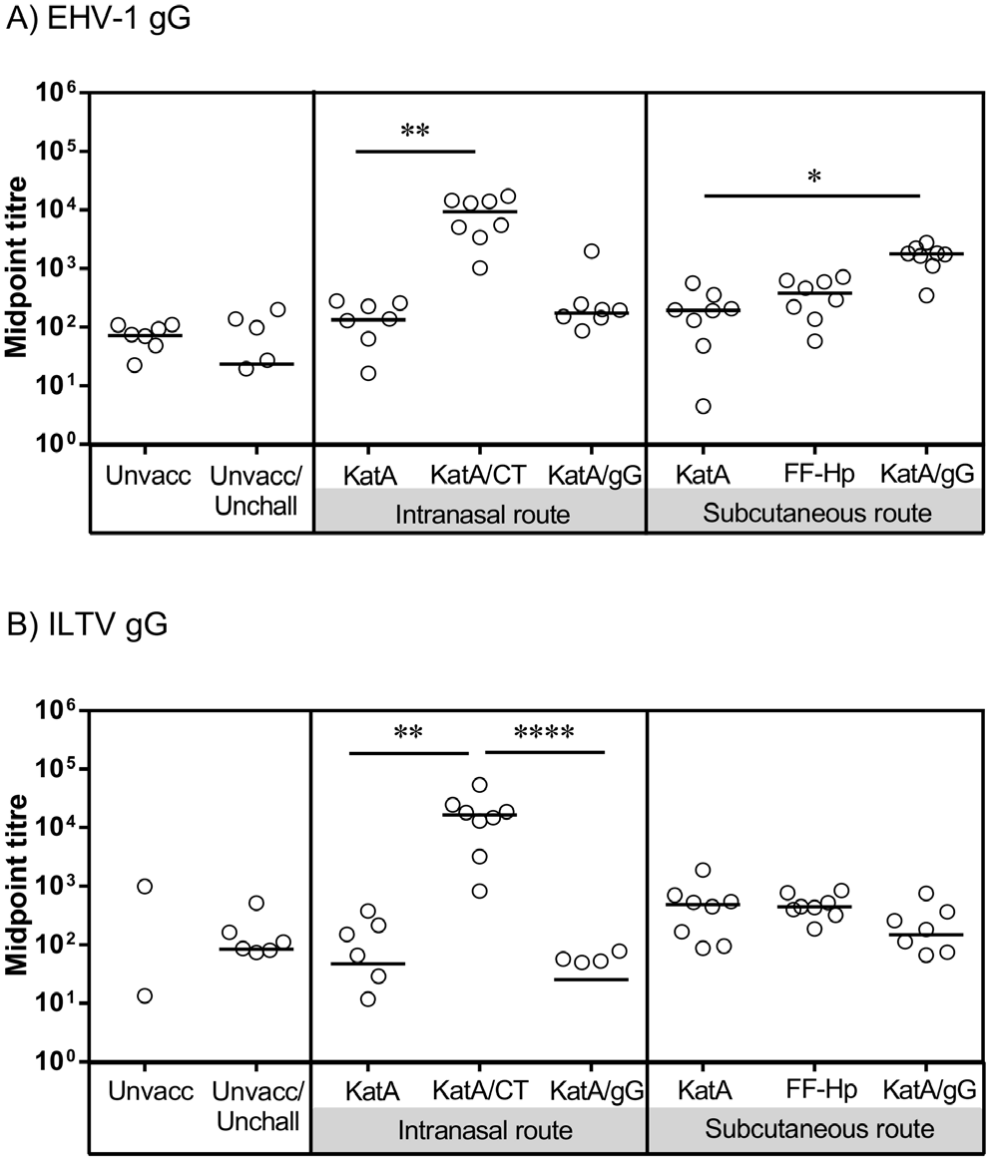
Immunisation with KatA and EHV-1 gG, but not ILTV gG, boosts specific antibody (IgG) production. BALB/c mice were remained unvaccinated (unvacc), or were vaccinated via the intranasal or subcutaneous route with either recombinant catalase (KatA) alone (10 µg), KatA (10 µg) adjuvanted with cholera toxin (CT, 10 µg, i.n. only), formalin-fixed *H. pylori* (FF-Hp, s.c. only) or KatA (10 µg) in combination with either A) EHV-1 gG (10 µg) or B) ILTV gG (10 µg). Two weeks after the second vaccination, tail bleeds were performed and anti-catalase IgG levels in serum were determined. Each point represents an individual mouse; the line represents the median. *p<0.05, ***p<0.0005, ****p<0.00005, Kruskal-Wallis with Dunn’s Multiple Comparison test. All other comparisons were not significant.

EHV-1 gG binds a broad range of murine and human chemokines (Bryant et al, 2003) suggesting that it has the capacity to modulate immune responses in multiple species. ILTV also has the capacity to bind murine and human chemokines, albeit a narrower range (Devlin et al, 2010). Consequently, we chose to test the immuno-modulatory potentials of EHV-1 and ILTV gG in an established murine vaccine/challenge model for *Helicobacter pylori*. We selected KatA as the vaccine antigen, which we and others have previously shown to induce protective immunity in mice when delivered with an appropriate adjuvant [31], [34], [35]. We co-delivered gG with catalase on two occasions, three weeks apart – a regimen that has proven effective in our laboratory [30], [31]. Serum IgG levels measured one week after the second vaccination indicated that, as expected, a strong antibody response was elicited in mice vaccinated with KatA and CT (Figure 3), which was significantly higher than in mice vaccinated with KatA alone (**p<0.01; Kruskal-Wallis with Dunn’s multiple comparison). Immunisation with KatA + gG (either EHV-1 or ILTV) via the intranasal route failed to boost IgG levels above those observed in mice vaccinated with KatA alone. Mice vaccinated with KatA + ILTV gG via the subcutaneous route also failed to exhibit a boosted antibody response. However, in mice vaccinated with KatA + gG via the subcutaneous route, serum IgG was significantly boosted when compared to mice vaccinated with KatA alone (*p<0.05; Kruskal-Wallis with Dunn’s multiple comparison). These data indicate that EHV-1 gG, but not ILTV gG, can boost immune responses to vaccine antigens, when delivered via the s.c. route.

### Mice Immunised with *H. Pylori* Catalase and EHV-1 gG, but not ILTV gG, have Reduced Bacterial Burden Following Challenge

**Figure 4:**
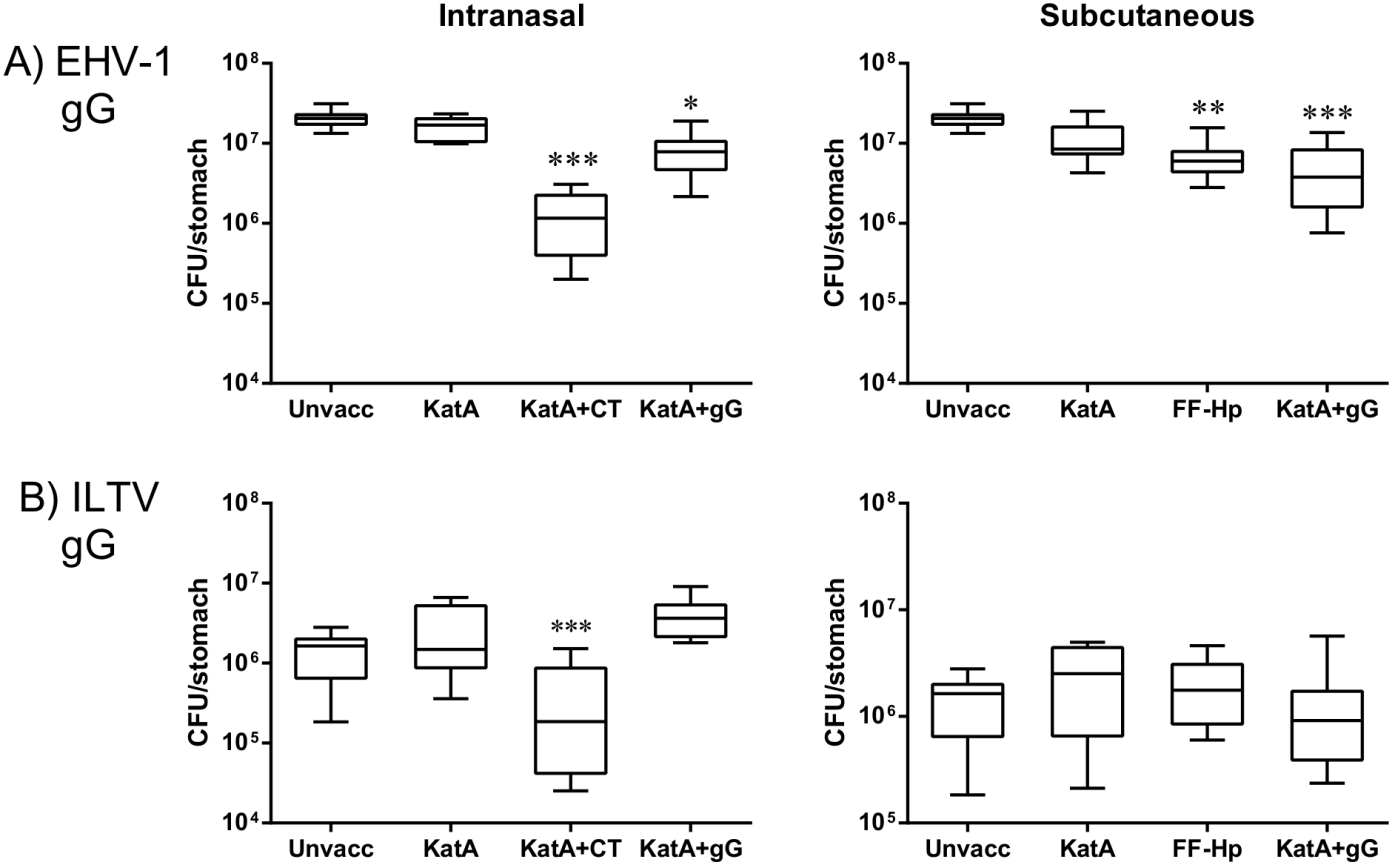
Immunisation with KatA and EHV-1 gG, but not ILTV gG, resulted in reduced *H. pylori* colonisation levels. BALB/c mice were not vaccinated (unvacc) or vaccinated via the i.n. route recombinant catalase (KatA) alone (10 µg), KatA + CT (10 µg of each) or KatA and either A) EHV-1 gG or B) ILTV gG (10 µg of each). Mice vaccinated subcutaneously received recombinant catalase (KatA) alone (10 µg), formalin-fixed *H. pylori* (FF-HP) or KatA and either A) EHV-1 gG or B) ILTV gG (10 µg of each), or were not vaccinated (unvacc). Four weeks after the second vaccination, mice were challenged with *H. pylori* (10^7^, oro-gastric delivery). Box plots represent the median (horizontal line), interquartile range (box) and the 10^th^ and 90^th^ percentiles (whiskers). *p<0.05, **p<0.005, ***p<0.0005, compared to unvaccinated group; Kruskal-Wallis with Dunn’s Multiple Comparison test.

Mice vaccinated with KatA alone did not have significantly reduced bacterial burdens after challenge when compared to unvaccinated mice, via either the i.n. or s.c. route (Figure 4). As anticipated mice that were vaccinated with KatA adjuvanted with CT i.n. or with formalin-fixed whole bacteria s.c. had significantly reduced bacterial burdens when compared to unvaccinated mice or mice vaccinated with KatA alone (Figure 4). Subcutaneous delivery of KatA + EHV-1 gG resulted in a highly significant reduction in bacterial load observed when compared to unvaccinated mice (***p<0.001). Mice vaccinated with KatA + EHV-1 gG via the i.n. route also showed a significant reduction in bacterial loads following challenge (Figure 4A, *p<0.05). In contrast, bacterial burdens in mice vaccinated with KatA + ILTV gG were not reduced (Figure 4B).

### Reduced Bacterial Burdens in Mice Immunised with *H. Pylori* Catalase and EHV-1 gG is not due to Post-immunisation Gastritis

Stomachs were examined histologically to determine whether increased generalised inflammation could account for the reduced bacterial burdens in mice immunised with *H. pylori* catalase and EHV-1 gG. All mice infected with *H. pylori* exhibited mild cellular infiltrate (Table 2). There was significantly more cellular infiltrate (p<0.01), mucus metaplasia (p<0.05) and atrophy (p<0.05) in mice that were vaccinated with *H. pylori* catalase and cholera toxin i.n. when compared with mice that were challenged, but unvaccinated. However, mice in the groups that were vaccinated with *H. pylori* catalase and EHV-1 gG did not show significantly more severe gastritis than mice that were unvaccinated/challenged.

**Table 2:** Histopathology scores in mice.

	Median gastritis scores^a^ (interquartile ranges)		
Vaccination	Cellular infiltrate	Mucus metaplasia	Atrophy
Unvaccinated/unchallenged	0 (0–1)	0 (0–0)	0 (0–0)
Unvaccinated/challenged	2 (1–2)	0 (0–0)	0 (0–0)
Catalase i.n.	0.5 (0–1.75)	0 (0–0)	0 (0–0)
Catalase + CT i.n.	3.5 (2.25–4)**	0 (0–0.75)*	0 (0–1)**
Catalase + EHV-1 gG i.n.	1.5 (1–2)	0 (0–0)	0 (0–0)
Catalase s.c.	2(1–2.75)	0 (0–0)	0 (0–0)
Formalin-fixed *H. pylori* s.c.	2 (2–2.75)	0 (0–0)	0 (0–0)
Catalase + EHV-1 gG s.c.	2.0 (2–3)	0 (0–0)	0 (0–0)

a Cellular infiltrate (0–6): 0, none; 1 mild, multifocal; 2 mild, widespread or moderate multifocal; 3, mild widespread and moderate multifocal or severe multifocal; 4, moderate widespread; 5, moderate widespread and severe multifocal; and 6, severe widespread. Mucus metaplasia and functional atrophy (0–3): 0, absent; 1 mild; 2 moderate; and 3 severe.

*p<0.05, **p<0.01, c.f. unvaccinated/challenged group; One-way ANOVA with Dunnett’s Multiple Comparison Test

## Discussion

This is the first *in vivo* investigation into the use of alphaherpesvirus gG to alter immune responses to a heterologous antigen. The results showed that EHV-1 gG, but not ILTV gG, enhanced protective immune responses to *H, pylori* KatA antigen.

In this study, our investigations confirmed that recombinant EHV-1 gG binds a broad range of chemokines, consistent with findings from previous studies [5], [7], [13]. Moreover, in addition to its broad-spectrum chemokine-binding ability, EHV-1 gG was also found to bind to the murine Th2 cytokine, IL-4. This is the first report of any species of viral glycoprotein G binding to a non-chemokine cytokine. We tested the capacity of EHV-1 gG and ILTV gG to modulate immune responses to a vaccine antigen in a mouse *H. pylori* vaccine/challenge model, and whether this could subsequently impact upon bacterial burdens. These Gram negative bacteria infect the stomachs of approximately half the world’s population, and are the major cause of gastric cancer. Although currently treated with a combination of two antibiotics and a proton pump inhibitor, like many bacterial diseases, the rising prevalence of antibiotic-resistant strains is of increasing concern. A recent multi-centre survey in Europe highlighted increasing prevalence of resistance to clarithromycin as well as the emergence of levofloxacin-resistant strains [36]. These concerning findings emphasise the need to develop novel therapeutics; vaccines represent the most cost-effective way of tackling this disease [37]. As another pathogen that has co-evolved with its human host over millennia [38], *H. pylori* has developed numerous strategies to evade host immune responses and subsequent clearance [37]. These adaptations are particularly evident when considering vaccination protocols tested in mice usually only result in a one- to two-log fold reduction in bacterial burdens at best, even when antigens are co-delivered with the ‘gold standard’ mucosal adjuvant, cholera toxin [30], [31]. We recently postulated that modulation of the inflammatory response through manipulation of the chemokine and cytokine milieu could represent a novel and improved approach to induce robust immune responses to *H. pylori* vaccine antigens and may subsequently improve vaccine efficacy [37]. A vCKBP has the potential to fulfil this role.

This study tested whether glycoprotein G derived from EHV-1 or ILTV could be used to modulate immune responses to recombinant *H. pylori* catalase and whether this would lead to protective immunity against *H. pylori* challenge. When delivered s.c., a significantly higher titred antibody response was induced in mice that received KatA plus EHV-1 gG than in mice that were immunised with KatA alone. An enhanced protection by way of reduced bacterial burdens was then detected following challenge. Although there was no detectable enhancement in the antibody response to KatA when co-delivered with EHV-1 gG via the i.n. route, a significant reduction in bacterial burden was observed when compared to mice that received KatA alone. Th1 immunity, rather than Th2 immunity is reportedly essential for *H. pylori* vaccine-mediated protection [39] and so it is not inconceivable that an enhanced level of protection was observed in the absence of an increased antibody response. Importantly, non-specific inflammation did not account for the reduction in bacterial burdens observed following immunisation with KatA + EHV-1 gG, which was no worse than the mild gastritis observed in unvaccinated/challenged mice. Gastritis is commonly associated with *H. pylori* vaccines [40] and was evident here in the KatA + CT i.n. group.

In contrast to EHV-1 gG, ILTV gG did not boost antibody responses to KatA and failed to effect any protection by way of reduced bacterial burdens. The differences between the *in vivo* effects of EHV-1 and ILTV gG in this model may be related to differences in their chemokine-binding profiles. EHV-1 gG is considered a broad-spectrum chemokine binding protein [5] but has only been tested against a limited range of murine chemokines. In previous studies EHV-1 gG has shown binding to murine chemokines from the CCL subfamily (CCL5, CCL11) and the CXL subfamily (CXCL1, CXCL2, CXCL9, CXCL13,) as well the murine chemokine XCL1. EHV-1 gG has not shown binding to murine chemokines CXCL10, CXCL12, CCL2, CCL3, CCL20, CCL21, CCL27 or CXC_3_L1 [5]. ILTV gG is considered to bind a narrower range of chemokines but has been tested against a greater range of murine chemokines. It has shown binding to murine chemokines in the CCL subfamily (CCL21, CCL24, CCL25, CCL27, CCL28) and the CXCL subfamily (CXCL11, CXCL12β, CXCL13, CXCL14) [7]. ILTV gG has not shown binding to murine chemokines CCL2, CCL3, CCL4, CCL5, CCL6, CCL7, CCL8, CCL9/10, CCL11, CCL12, CCL17, CCL19, CCL20, CXCL1, CXCL2, CXCL4, CXCL5, CXCL9, CXCL15 or CXCL16 [7]. Future studies to more comprehensively investigate the breadth and strength of EHV-1 gG binding to chemokines, similar to those performed for ILTV gG [7] are indicated. Interestingly, previous studies have suggested that ILTV gG may act to skew the host immune response towards a Th2-mediated response [7] but a similar role for EHV-1 gG has not been described [41]. These differences could be related to the different effects of EHV-1 and ILTV gG in this model, which requires Th1 immune responses for protection. Clarifying whether EHV-1 gG chemokine-binding with subsequent modulation of T-cell responses is the mechanism responsible for the protective immunity observed is of utmost priority in future studies to reach a full understanding of how gG can enhance immunity.

The findings of this study present a promising avenue for development of novel vaccine adjuvants and warrants further investigation, which should focus on the mechanism of action. Moreover, by increasing our understanding of vCKBP interaction with chemokines and cytokines, we envisage the potential to develop tailored adjuvants with specific chemokine-binding profiles that can then be utilised for highly specific vaccines. This technology has potential applications not just for development of efficacious *H. pylori* vaccines, but vaccines for other infectious diseases.

## References

[1] Alcami A, Lira SA (2010) Modulation of chemokine activity by viruses. Curr Opin Immunol 22: 482–487. 20598516 10.1016/j.coi.2010.06.004 PMC6373461

[2] Alcami A, Saraiva M (2009) Chemokine binding proteins encoded by pathogens. Adv Exp Med Biol 666: 167–179. 20054983 10.1007/978-1-4419-1601-3_13

[3] Webb LM, Alcami A (2005) Virally encoded chemokine binding proteins. Mini Rev Med Chem 5: 833–848. 16178725 10.2174/1389557054867110

[4] Viejo-Borbolla A, Martinez-Martin N, Nel HJ, Rueda P, Martin R, et al (2012) Enhancement of chemokine function as an immunomodulatory strategy employed by human herpesviruses. PLoS Pathog 8: e1002497. 22319442 10.1371/journal.ppat.1002497 PMC3271085

[5] Bryant NA, Davis-Poynter N, Vanderplasschen A, Alcami A (2003) Glycoprotein G isoforms from some alphaherpesviruses function as broad-spectrum chemokine binding proteins. Embo J 22: 833–846. 12574120 10.1093/emboj/cdg092 PMC145452

[6] Costes B, Thirion M, Dewals B, Mast J, Ackermann M, et al (2006) Felid herpesvirus 1 glycoprotein G is a structural protein that mediates the binding of chemokines on the viral envelope. Microbes Infect 8: 2657–2667. 16962359 10.1016/j.micinf.2006.07.014

[7] Devlin JM, Viejo-Borbolla A, Browning GF, Noormohammadi AH, Gilkerson JR, et al (2010) Evaluation of immunological responses to a glycoprotein G deficient candidate vaccine strain of infectious laryngotracheitis virus. Vaccine 28: 1325–1332. 19932672 10.1016/j.vaccine.2009.11.013

[8] Fallon PG, Alcami A (2006) Pathogen-derived immunomodulatory molecules: future immunotherapeutics? Trends Immunol 27: 470–476. 16920025 10.1016/j.it.2006.08.002

[9] Lucas A, McFadden G (2004) Secreted immunomodulatory viral proteins as novel biotherapeutics. J Immunol 173: 4765–4774. 15470015 10.4049/jimmunol.173.8.4765

[10] Luster AD, Alon R, von Andrian UH (2005) Immune cell migration in inflammation: present and future therapeutic targets. Nat Immunol 6: 1182–1190. 16369557 10.1038/ni1275

[11] Proudfoot AE (2002) Chemokine receptors: multifaceted therapeutic targets. Nat Rev Immunol 2: 106–115. 11910892 10.1038/nri722 PMC7097668

[12] Wells TN, Power CA, Shaw JP, Proudfoot AE (2006) Chemokine blockers–therapeutics in the making? Trends Pharmacol Sci 27: 41–47. 16310864 10.1016/j.tips.2005.11.001

[13] Van de Walle GR, May ML, Sukhumavasi W, von Einem J, Osterrieder N (2007) Herpesvirus chemokine-binding glycoprotein G (gG) efficiently inhibits neutrophil chemotaxis *in vitro* and *in vivo* . J Immunol 179: 4161–4169. 17785855 10.4049/jimmunol.179.6.4161

[14] Viejo-Borbolla A, Muñoz A, Tabarés E, Alcamí A (2010) Glycoprotein G from pseudorabies virus binds to chemokines with high affinity and inhibits their function. J Gen Virol 91: 23–31. 19776237 10.1099/vir.0.011940-0

[15] Devlin JM, Browning GF, Gilkerson JR, Fenton SP, Hartley CA (2008) Comparison of the safety and protective efficacy of vaccination with glycoprotein-G-deficient infectious laryngotracheitis virus delivered via eye-drop, drinking water or aerosol. Avian Pathol 37: 83–88. 18202954 10.1080/03079450701802214

[16] Devlin JM, Browning GF, Hartley CA, Gilkerson JR (2007) Glycoprotein G deficient infectious laryngotracheitis virus is a candidate attenuated vaccine. Vaccine 25: 3561–3566. 17316926 10.1016/j.vaccine.2007.01.080

[17] Devlin JM, Hartley CA, Gilkerson JR, Coppo MJ, Vaz P, et al (2011) Horizontal transmission dynamics of a glycoprotein G deficient candidate vaccine strain of infectious laryngotracheitis virus and the effect of vaccination on transmission of virulent virus. Vaccine 29: 5699–5704. 21689710 10.1016/j.vaccine.2011.06.002

[18] Dabbagh K, Xiao Y, Smith C, Stepick-Biek P, Kim SG, et al (2000) Local Blockade of Allergic Airway Hyperreactivity and Inflammation by the Poxvirus-Derived Pan-CC-Chemokine Inhibitor vCCI. J Immunol 165: 3418–3422. 10975861 10.4049/jimmunol.165.6.3418

[19] Liu L, Lalani A, Dai E, Seet B, Macauley C, et al (2000) The viral anti-inflammatory chemokine-binding protein M-T7 reduces intimal hyperplasia after vascular injury. J Clin Invest 105: 1613–1621. 10841520 10.1172/JCI8934 PMC300852

[20] Czinn SJ, Blanchard T (2011) Vaccinating against *Helicobacter pylori* infection. Nat Rev Gastroenterol Hepatol 8: 133–140. 21304478 10.1038/nrgastro.2011.1

[21] Michetti P, Kreiss C, Kotloff KL, Porta N, Blanco JL, et al (1999) Oral immunization with urease and *Escherichia coli* heat-labile enterotoxin is safe and immunogenic in *Helicobacter pylori*-infected adults. Gastroenterology 116: 804–812. 10092302 10.1016/s0016-5085(99)70063-6

[22] Banerjee S, Medina-Fatimi A, Nichols R, Tendler D, Michetti M, et al (2002) Safety and efficacy of low dose *Escherichia coli* enterotoxin adjuvant for urease based oral immunisation against *Helicobacter pylori* in healthy volunteers. Gut 51: 634–640. 12377799 10.1136/gut.51.5.634 PMC1773429

[23] Sougioultzis S, Lee CK, Alsahli M, Banerjee S, Cadoz M, et al (2002) Safety and efficacy of *E. coli* enterotoxin adjuvant for urease-based rectal immunization against *Helicobacter pylori* . Vaccine 21: 194–201. 12450694 10.1016/s0264-410x(02)00467-x

[24] Aebischer T, Bumann D, Epple HJ, Metzger W, Schneider T, et al (2008) Correlation of T cell response and bacterial clearance in human volunteers challenged with *Helicobacter pylori* revealed by randomised controlled vaccination with Ty21a-based *Salmonella* vaccines. Gut 57: 1065–1072. 18417532 10.1136/gut.2007.145839 PMC2564837

[25] Angelakopoulos H, Hohmann EL (2000) Pilot study of phoP/phoQ-deleted *Salmonella enterica* serovar typhimurium expressing *Helicobacter pylori* urease in adult volunteers. Infect Immun 68: 2135–2141. 10722611 10.1128/iai.68.4.2135-2141.2000 PMC97395

[26] Malfertheiner P, Schultze V, Rosenkranz B, Kaufmann SH, Ulrichs T, et al (2008) Safety and immunogenicity of an intramuscular *Helicobacter pylori* vaccine in noninfected volunteers: a phase I study. Gastroenterology 135: 787–795. 18619971 10.1053/j.gastro.2008.05.054

[27] Studdert MJ, Blackney MH (1979) Equine herpesviruses: on the differentiation of respiratory from foetal strains of type 1. Aust Vet J 55: 488–492. 231960 10.1111/j.1751-0813.1979.tb00377.x

[28] Drummer HE, Studdert MJ, Crabb BS (1998) Equine herpesvirus-4 glycoprotein G is secreted as a disulphide-linked homodimer and is present as two homodimeric species in the virion. J Gen Virol 79 (Pt 5): 1205–1213. 10.1099/0022-1317-79-5-1205 9603336

[29] Lee A, O’Rourke J, De Ungria MC, Robertson B, Daskalopoulos G, et al (1997) A standardized mouse model of *Helicobacter pylori* infection: introducing the Sydney strain. Gastroenterology 112: 1386–1397. 9098027 10.1016/s0016-5085(97)70155-0

[30] Every AL, Stent A, Moloney MB, Ng GZ, Skene CD, et al (2011) Evaluation of superoxide dismutase from *Helicobacter pylori* as a protective vaccine antigen. Vaccine 29: 1514–1518. 21172379 10.1016/j.vaccine.2010.12.019

[31] Stent A, Every AL, Ng GZ, Chionh YT, Ong LS, et al (2012) *Helicobacter pylori* thiolperoxidase as a protective antigen in single- and multi-component vaccines. Vaccine 30: 7214–7220. 23084846 10.1016/j.vaccine.2012.10.022

[32] Every AL, Ng GZ, Skene CD, Harbour SN, Walduck AK, et al (2011) Localized suppression of inflammation at sites of *Helicobacter pylori* colonization. Infect Immun 79: 4186–4192. 21807907 10.1128/IAI.05602-11 PMC3187257

[33] Van de Walle GR, Kaufer BB, Chbab N, Osterrieder N (2009) Analysis of the herpesvirus chemokine-binding glycoprotein G residues essential for chemokine binding and biological activity. J Biol Chem 284: 5968–5976. 19074431 10.1074/jbc.M808127200

[34] Radcliff FJ, Hazell SL, Kolesnikow T, Doidge C, Lee A (1997) Catalase, a novel antigen for *Helicobacter pylori* vaccination. Infect Immun 65: 4668–4674. 9353048 10.1128/iai.65.11.4668-4674.1997 PMC175669

[35] Skene CD, Doidge C, Sutton P (2008) Evaluation of ISCOMATRIX and ISCOM vaccines for immunisation against *Helicobacter pylori* . Vaccine 26: 3880–3884. 18547687 10.1016/j.vaccine.2008.05.004

[36] Megraud F, Coenen S, Versporten A, Kist M, Lopez-Brea M, et al (2013) *Helicobacter pylori* resistance to antibiotics in Europe and its relationship to antibiotic consumption. Gut 62: 34–42. 22580412 10.1136/gutjnl-2012-302254

[37] Every AL (2013) Key host–pathogen interactions for designing novel interventions against *Helicobacter pylori* . Trends Microbiol 21: 253–259. 23528348 10.1016/j.tim.2013.02.007

[38] Covacci A, Telford JL, Del Giudice G, Parsonnet J, Rappuoli R (1999) *Helicobacter pylori* virulence and genetic geography. Science 284: 1328–1333. 10334982 10.1126/science.284.5418.1328

[39] Sayi A, Kohler E, Hitzler I, Arnold I, Schwendener R, et al (2009) The CD4+ T cell-mediated IFN-gamma response to *Helicobacter* infection is essential for clearance and determines gastric cancer risk. J Immunol 182: 7085–7101. 19454706 10.4049/jimmunol.0803293

[40] Sutton P, Danon SJ, Walker M, Thompson LJ, Wilson J, et al (2001) Post-immunisation gastritis and *Helicobacter* infection in the mouse: a long term study. Gut 49: 467–473. 11559641 10.1136/gut.49.4.467 PMC1728471

[41] Van de Walle GR, Jarosinski KW, Osterrieder N (2008) Alphaherpesviruses and chemokines: pas de deux not yet brought to perfection. J Virol 82: 6090–6097. 18385255 10.1128/JVI.00098-08 PMC2447056

